# Serum vitamin D levels and prostate cancer: an umbrella review and pooled analysis of observational meta-analyses

**DOI:** 10.3389/fonc.2026.1798697

**Published:** 2026-06-29

**Authors:** Li Wei, Lina Fan, Huifen Wang

**Affiliations:** Department of Urology, The Affiliated Yangming Hospital of Ningbo University, Yuyao, Zhejiang, China

**Keywords:** cancer risk, meta-analysis, observational studies, prostate cancer, umbrella review, vitamin D

## Abstract

**Background:**

This umbrella review examines the controversial link between serum vitamin D levels and prostate cancer (PC) risk by systematically re-analyzing existing meta-analyses. It aims to synthesize the available evidence while acknowledging the inherent limitations of umbrella reviews and observational data.

**Methods:**

A systematic search of PubMed, Scopus, and Web of Science was performed to identify all meta-analyses assessing serum vitamin D levels and PC risk up to January 2025. Eligible studies were observational meta-analyses reporting pooled effect sizes (ORs, RRs, or HRs) to determine the association between circulating vitamin D and PC incidence; however, other types of designs were excluded. Data extraction was performed using a standardized framework. Heterogeneity assessment was performed by utilizing Cochran’s Q test and I^2^ statistics; Re-analysis of extracted data was conducted using random-effects models, and leave-one-out sensitivity analyses evaluated the stability of pooled associations.

**Results:**

Pooled categorical analyses suggested a small but statistically significant association between higher serum vitamin D levels and increased PC risk (OR: 1.06; 95% CI: 1.02–1.09; p=0.001), with low heterogeneity. Moreover, sensitivity analyses showed consistent findings across individual study exclusions. In contrast, pooled relative risk estimates per 10-ng/mL increment in serum vitamin D did not show a significant association (RR: 1.02; 95% CI: 0.99–1.06; p=0.207). Sensitivity analyses did not materially change these findings.

**Conclusion:**

Higher serum vitamin D levels may be associated with a slightly increased odds of PC in categorical analyses, but the evidence does not support a clear linear dose–response relationship. Given the observational nature of the included evidence, residual confounding, and modest effect sizes, these findings should be interpreted cautiously.

## 1 Introduction

Prostate cancer (PC) remains one of the leading malignancies among men worldwide and continues to impose a profound burden on health systems despite major advances in screening and treatment strategies (1, 2). Identifying determinants of prostate carcinogenesis has therefore become a priority in cancer prevention research (3). Among the factors that have attracted scientific attention, serum vitamin D status has emerged as a potential contributor to PC development (4). Vitamin D, synthesized predominantly through skin exposure to ultraviolet B radiation, plays critical roles in numerous metabolic pathways, like immune regulation, cellular differentiation, etc. (5, 6),. In addition, Dwivedi et al. (2025) (7) declared that vitamin D plausibly impacts on PC through VDR signaling modulation, androgen regulation and lowering inflammation (7). On the other hand, vitamin D could increase expression of CYP24A1 which may negatively reduce vitamin D activity in PC patients (8). These biological functions have given rise to the long-standing hypothesis that vitamin D may influence cancer initiation and progression (6).

Over the past two decades, observational evidence examining the relationship between circulating vitamin D concentrations and PC risk has expanded rapidly (9). However, despite the biological plausibility and widespread clinical interest, epidemiological findings have been highly inconsistent. Some studies have suggested that low serum vitamin D levels may increase PC susceptibility, whereas others have reported no association or have even indicated an elevated risk with higher vitamin D concentrations (4, 9, 10). These conflicting results have led to numerous meta-analyses, which themselves differ in methodological rigor, for example, dose-response design (4), OR calculation (9), or RR estimation (10), exposure definitions, statistical approaches, and study populations, making it challenging to derive clear conclusions.

As one of the highest tiers in the evidence hierarchy for observational associations, umbrella reviews may provide an opportunity to comprehensively evaluate the consistency, robustness, and credibility of findings across multiple meta-analyses (11). Given the global prevalence of vitamin D deficiency and the widespread use of vitamin D supplementation—often exceeding recommended levels—the question of whether circulating vitamin D influences PC risk has substantial implications for both clinical practice and public health policy (12). A rigorous synthesis of existing meta-analytic evidence is therefore urgently needed.

The present umbrella review was designed to address this gap by systematically identifying all available observational meta-analyses on serum vitamin D and PC incidence and re-analyzing their result integrally to update pooled estimates, conducting sensitivity analyses, and comparing categorical and continuous exposure measures. This study aims to clarify the nature of the association and to provide a more reliable foundation for future research priorities and clinical decision-making.

## 2 Materials and methods

This umbrella review was conducted to systematically identify, evaluate, and re-analyze observational meta-analyses that examined the relationship between circulating vitamin D biomarkers and the incidence of PC. Although the protocol of this study was not registered, this paper followed PRISMA guidelines and adhered to established methodological standards for umbrella reviews synthesizing epidemiological evidence (13).

### 2.1 Literature search strategy

A comprehensive and systematic search of PubMed, Scopus, and Web of Science was conducted from database inception through January 2025. Search strategies combined controlled vocabulary and free-text terms related to vitamin D biomarkers (“circulating 25-hydroxyvitamin D,” “serum 25(OH)D,” “circulating 1,25-dihydroxyvitamin D,” “1,25(OH)_2_D level,” “serum vitamin D”), PC (“prostate neoplasm,” “prostate carcinoma,” “cancer incidence”), and meta-analytic designs (“systematic review,” “meta-analysis,” “pooled analysis”). The search strategy details can be found in **Supplementary Table 1**. No restrictions were applied to language, location, or publication year. Reference lists of eligible articles and previous reviews were systematically screened to identify any additional meta-analyses.

### 2.2 Eligibility criteria

Meta-analyses were eligible if they applied a systematic review methodology with quantitative meta-analyses, synthesized observational studies (cohort, case–control, or nested case–control), assessed at least one circulating vitamin D biomarker (e.g., serum/plasma 25(OH)D or 1,25(OH)_2_D), reported PC incidence as the primary outcome, and provided pooled effect sizes (ORs, RRs, or HRs with 95% CIs); conversely, meta-analyses of supplementation trials, those focusing solely on dietary intake or sunlight exposure due to lack of considering all affecting factors on circulating vitamin D, narrative reviews, and studies without extractable quantitative data were excluded.

### 2.3 Study selection and data extraction process

Two reviewers (LW, LF) independently screened titles and abstracts of all retrieved records. Full texts of potentially relevant articles were then assessed for eligibility. Discrepancies at any stage were resolved through discussion or consultation with a third reviewer (HW). Data extraction was conducted independently by two reviewers (LW, LF) using a predefined extraction template. Extracted variables included publication details, assay method, geographic region, number of included primary studies, biomarker type, exposure classification (categorical vs continuous), population characteristics, statistical models (fixed or random effects), and pooled effect estimates. When overlapping primary studies were identified across included meta-analyses, we prioritized the most comprehensive and methodologically robust synthesis to minimize double-counting of evidence. A citation matrix was consulted, and where substantial overlap existed (>50% corrected covered area), only the highest-quality meta-analysis was retained for primary analysis; this occurred infrequently due to heterogeneity in exposure definitions and study designs.

### 2.4 Statistical analysis

The pooled effect size and its 95% CIs were calculated using a random-effects model with the restricted maximum likelihood (REML) method (14). For transparency, we clarify that we did not re-analyze individual patient data or original study-level data from primary studies. Instead, we extracted the published pooled effect sizes (ORs, RRs, or HRs) directly from each eligible meta-analysis and used these summary estimates as the input data for our own quantitative synthesis. Because the included meta-analyses reported effect estimates using different analytical approaches, two separate syntheses were performed. Categorical comparisons of the highest versus lowest vitamin D levels were summarized using ORs, whereas dose–response analyses evaluating the association per 10 ng/mL increment in circulating 25(OH)D were summarized using RRs. Heterogeneity was assessed using Cochran’s Q test and the I² statistic. Values of I² greater than 50% were considered indicative of moderate to substantial heterogeneity, prompting cautious interpretation of pooled estimates (15). Leave-one-out sensitivity analyses were conducted to evaluate the influence of each individual study on the pooled estimates. Due to a lower number of included observations, Subgroup and publication bias analyses were not performed. Statistical analyses were completed using STATA version 16 software (Stata Corp, College Station, Texas, USA).

### 2.5 Evaluating methodology and quality of evidence

Two researchers (LW, LF) independently assessed the methodological quality of the included studies using the AMSTAR2 assessment tool (16), which consists of 16 criteria with responses such as ‘yes,’ ‘partial yes,’ ‘no,’ or ‘no meta-analysis.’ Any disagreements were resolved through consensus between the two researchers or with the involvement of a third author (HW) if necessary. The AMSTAR 2 checklist classifies studies into four levels: ‘Critically low quality,’ ‘low quality,’ ‘moderate quality,’ and ‘high quality’.

## 3 Results

### 3.1 Literature search

**Figure 1:**
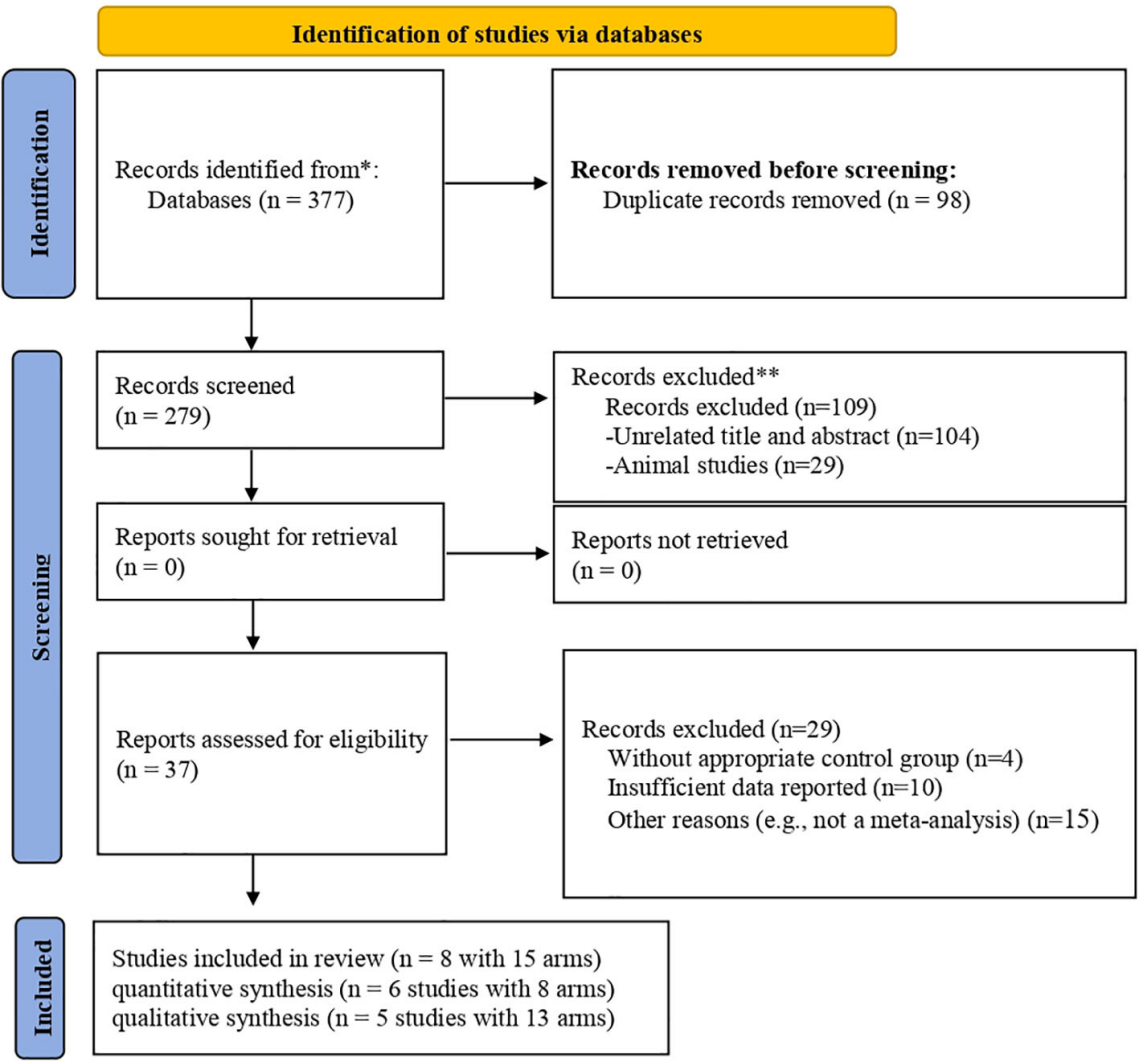
PRISMA flow diagram showing the selection process of included meta-analyses. * publications from the databases considered, ** after considering the eligibility criteria described in the text.

**Figure 1** shows the flow of our study selection process. We initially identified 377 articles through our database search. After removing 98 duplicates, 279 unique articles remained. The titles and abstracts of these articles were screened, and 242 of them, were excluded leaving 37 for a full-text review. Ultimately, 8 studies including 15 arms (effect sizes) met our inclusion criteria and were selected in this study. The selection process flow diagram is illustrated in **Figure 1**.

### 3.2 Characteristics of included meta-analyses

The characteristics of the 15 analytical arms are summarized in **Table 1**. The included meta-analyses synthesized data from 5 to 21 primary observational studies each, with sample sizes ranging from 1,361 to 35,583 participants. The biomarker assessed was predominantly circulating 25-hydroxyvitamin D (25(OH)D), evaluated in 13 analytical arms, while two arms from one meta-analysis examined 1,25-dihydroxyvitamin D (1,25(OH)_2_D). Exposure contrasts were either categorical (e.g., high vs. low levels) or continuous (per 10 or 20 ng/mL increase). The most frequently reported effect measures were odds ratios (ORs) and relative risks (RRs). Six studies (with 8 analytical arms) were included in the quantitative synthesis (meta-analysis): Gilbert (2011) (17), Xu (2014) (9), Ghaderi (2024) (18), Yin (2009) (19), Gandini (2011) (10), and Gao (2018) (4). The remaining 2 studies (with 7 analytical arms) were included in the qualitative synthesis only: Song (2018) (20) and Cui (2024) (21).

**Table 1:** Characteristics of the 15 analytical arms included in the umbrella review.

Study	Sample size	N studies	Vitamin D biomarker	Exposure contrast	Statistical model	Effect size type	Outcome	Effect size (95% CI)	Study type
Gilbert, 2011 (a) (17)	4842	14	25(OH)D	High vs. Low	Random-effects	OR	cancer risk	1.04 (0.99–1.10)	Prospective Cohort
Gilbert, 2011 (b) (17)	1361	7	1,25(OH)_2_D	High vs. Low	Fixed-effects	OR	cancer risk	1.00 (0.87–1.14)	Prospective Cohort
Gilbert, 2011 (c) (17)	1361	7	1,25(OH)_2_D	High vs. Low	Random-effects	OR	cancer risk	1.00 (0.92–1.10)	Prospective Cohort
Ghaderi, 2024 (a) (18)	Not report	17	25(OH)D	High vs. Low	Random-effects	OR	cancer risk	1.06 (0.98–1.14)	Prospective Cohort
Ghaderi, 2024 (b) (18)	Not report	19	25(OH)D	High vs. Low	Fixed-effects	OR	cancer risk	1.06 (0.99–1.14)	Prospective Cohort
Xu, 2014 (a) (9)	19944	21	25(OH)D	Highest vs. Lowest	Random-effects	OR	cancer risk	1.17 (1.05–1.30)	Prospective Cohorts
Xu, 2014 (b) (9)	19944	21	25(OH)D	High vs. Low	Fixed-effects	OR	cancer risk	1.17 (1.08–1.27)	Prospective Cohorts
Xu, 2014 (c) (9)	13868	17	25(OH)D	Highest vs. Lowest	Random-effects	OR	cancer risk	1.18 (1.07–1.30)	Nested Case-Control
Xu, 2014 (d) (9)	13868	17	25(OH)D	High vs. Low	Fixed-effects	OR	cancer risk	1.17 (1.08–1.27)	Nested Case-Control
Yin, 2009 (a) (19)	7809	11	25(OH)D	Continuous (Per 10 ng/mL increase)	Fixed-effects	RR	cancer risk	1.03 (0.98–1.09)	Observational Studies
Yin, 2009 (b) (19)	7809	11	25(OH)D	Continuous (Per 10 ng/mL increase)	Random-effects	RR	cancer risk	1.03 (0.96–1.10)	Observational Studies
Gandini, 2011 (10)	22619	11	25(OH)D	Continuous (Per 10 ng/mL increase)	Random-effects	RR	cancer risk	0.99 (0.95–1.03)	Observational Studies
Gao, 2018 (4)	35583	19	25(OH)D	Continuous (Per 10 ng/mL increase)- High vs. Low	Random-effects	RR	cancer risk	1.04 (1.02–1.06) 1.15 (1.06–1.24)	Nested Case-Control
Song, 2018 (20)	5332	5	25(OH)D	Continuous (Per 20 ng/mL increase)	Random-effects	HR	Cancer mortality	0.91 (0.85–0.97)	Cohort Studies

### 3.3 Quality assessment

According to the AMSTAR-2 tool, the methodological quality of the included meta-analyses was rated as high for three studies (18, 19, 21), moderate for three studies (9, 10, 20), and low for two studies (4, 17) (**Table 2**). None was rated as critically low.

**Table 2:** AMSTAR-2 quality assessment of included meta-analyses.

Reference	Q1	Q2	Q3	Q4	Q5	Q6	Q7	Q8	Q9	Q10	Q11	Q12	Q13	Q14	Q15	Q16	Quality evaluation
Gandini,2011 (10)	No	Yes	Yes	Yes	NR	Yes	Yes	Yes	Yes	No	Partially yes	No	No	yes	Yes	Yes	Moderate
Xu, 2014 (9)	Yes	Yes	Yes	yes	NR	Yes	Yes	Yes	Yes	No	Partially yes	No	No	Yes	Yes	Yes	Moderate
Yin,2009 (19)	Yes	Yes	Yes	Partially yes	Yes	Yes	Yes	Yes	Yes	No	Yes	No	No	Yes	Yes	Yes	High
Gilbert, 2011 (17)	No	No	Yes	Yes	NR	Yes	Yes	Yes	Yes	No	Partially yes	No	No	Yes	Yes	Yes	Low
Song, 2018 (20)	No	Yes	Yes	Yes	NR	Yes	Yes	Yes	Yes	Yes	Yes	No	No	Yes	Yes	Yes	Moderate
Gao, 2018 (4)	No	Yes	Yes	yes	NR	Yes	Partially yes	Yes	Partially Yes	No	Yes	No	No	Yes	Yes	Yes	Low
Ghaderi,2024 (18)	Yes	Yes	Yes	Yes	NR	Yes	Yes	Yes	Yes	Yes	Yes	No	No	Yes	Yes	Yes	High
Cui, 2024 (21)	Yes	Yes	Yes	yes	Yes	Yes	Yes	Yes	Yes	Yes	Yes	No	No	Yes	Yes	Yes	High

1. Did the research questions and inclusion criteria for the review include the components of PICO? 2. Did the report of the review contain an explicit statement that the review methods were established prior to the conduct of the review and did the report justify any significant deviations from the protocol? 3. Did the review authors explain their selection of the study designs for inclusion in the review? 4. Did the review authors use a comprehensive literature search strategy? 5. Did the review authors perform study selection in duplicate? 6. Did the review authors perform data extraction in duplicate? 7. Did the review authors provide a list of excluded studies and justify the exclusions? 8. Did the review authors describe the included studies in adequate detail? 9. Did the review authors use a satisfactory technique for assessing the risk of bias (RoB) in individual studies that were included in the review? 10. Did the review authors report on the sources of funding for the studies included in the review? 11. If meta-analysis was performed, did the review authors use appropriate methods for statistical combination of results? 12. If meta-analysis was performed, did the review authors assess the potential impact of RoB in individual studies on the results of the meta-analysis or other evidence synthesis? 13. Did the review authors account for RoB in individual studies when interpreting/discussing the results of the review? 14. Did the review authors provide a satisfactory explanation for, and discussion of, any heterogeneity observed in the results of the review? 15. If they performed quantitative synthesis, did the review authors carry out an adequate investigation of publication bias (small study bias) and discuss its likely impact on the results of the review? 16. Did the review authors report any potential sources of conflict of interest, including any funding they received for conducting the review? Each question was answered with “Yes”, “Partial Yes” or “No”. When no meta-analysis was done, question 11, 12, and 15 were answered with “No meta-analysis conducted.”.

### 3.4 Results obtained from quantitative synthesis

#### 3.4.1 Association of vitamin D with prostate cancer based on OR (highest versus lowest levels of vitamin D)

**Figure 2:**
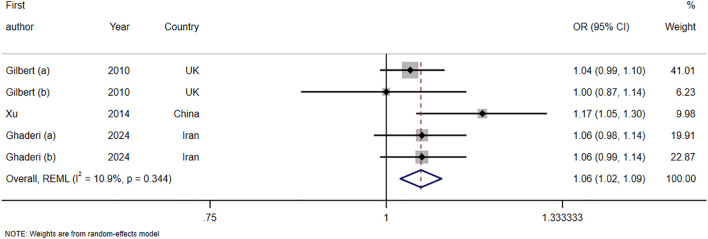
Forest plot of the association of vitamin D with prostate cancer based on OR (highest versus lowest levels of vitamin D).

Three studies with five analytical arms contributed to the categorical meta-analysis, which compared the highest versus lowest categories of vitamin D levels (9, 17, 18). The analysis showed a modest correlation between a rise in PC risk and higher circulating vitamin D levels, with a pooled effect of OR = 1.06 (95% CI: 1.02–1.09, p= 0.001), with low heterogeneity (I²=10.9%, P = 0.344) (**Figure 2**) (9, 17, 18). Sensitivity analyses demonstrated that the association was highly stable, with all leave-one-out estimates remaining above 1.00 and statistically significant. Excluding individual studies shifted the pooled effect only slightly, ranging from 1.046 to 1.071, and all confidence intervals remained clearly above unity (**Supplementary Figure 1**).

#### 3.4.2 Association of vitamin D with prostate cancer based on RR (per 10 ng/mL increase in serum 25(OH)D levels)

**Figure 3:**
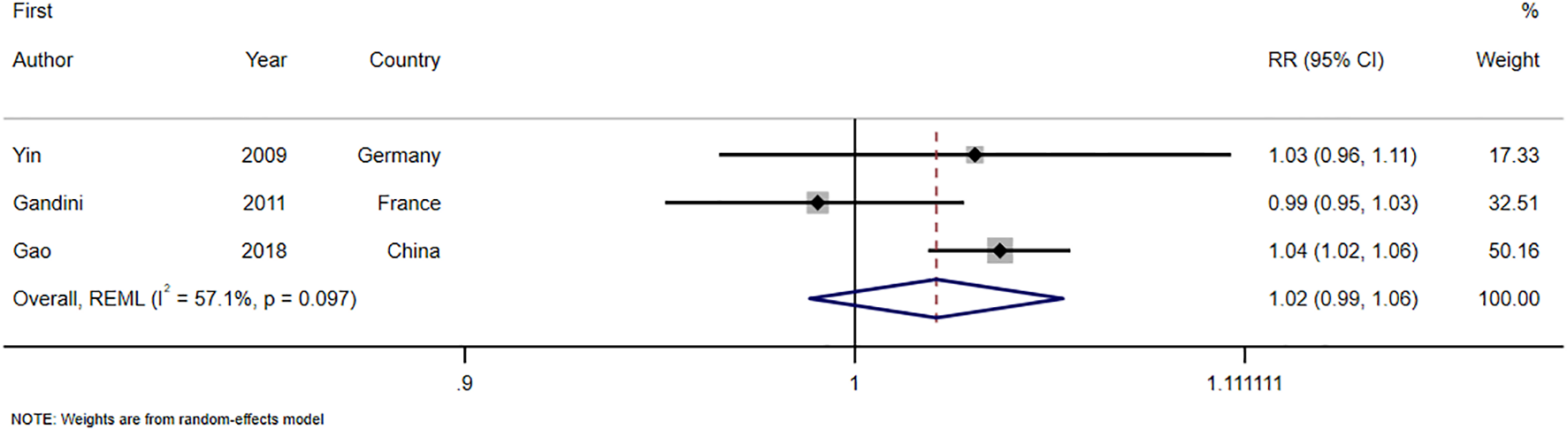
Forest plot of association of vitamin D with prostate cancer based on RR [per 10 ng/mL increase in serum 25(OH)D levels].

Three studies with three analytical arms contributed to the continuous dose–response meta-analysis, which evaluated the association per 10 ng/mL increase in serum 25(OH)D levels (4, 10, 19). The analysis did not show a statistically significant association, with a pooled estimate of RR = 1.02 (95% CI: 0.99–1.06, p=0.207) per 10 ng/mL increment, with moderate heterogeneity (I²=57.1%, P = 0.097) that illustrates results across studies have incoherent effect sizes (**Figure 3**). No significant effect was observed following sensitivity analysis (**Supplementary Figure 2**).

### 3.5 Results obtained from qualitative assessment

#### 3.5.1 Evidence for the active vitamin D metabolite

Evidence for the active hormonal metabolite, 1,25(OH)_2_D, was limited to one study (Gilbert et al., 2011) (17) comprising three analytical arms (17), all demonstrated null associations, including OR = 1.00 (0.87–1.14) and OR = 1.00 (0.92–1.10) across fixed- and random-effects models. These findings suggest that short-term hormonal fluctuations in vitamin D activity do not meaningfully contribute to PC risk and that long-term vitamin D stores, reflected by 25(OH)D, are more relevant to carcinogenic pathways.

#### 3.5.2 Association of vitamin D with prostate cancer incidence based on OR effect sizes

Three studies (seven analytical arms) reporting ORs were included to evaluate the association between circulating 25(OH)D levels and PC incidence (9, 17, 18). Among these, two arms—specifically Xu, 2014 (c) (9) and Xu, 2014 (d) (9)—were derived from a nested case–control design with pre-diagnostic vitamin D assessment.

Xu et al. (2014) (9) reported results using both statistical models. In both random-effects analysis (Xu 2014 a) (9) and fixed-effects analysis (Xu 2014 b) (9), the association was confirmed (OR = 1.17; 95% CI: 1.05–1.30) and (OR = 1.18; 95% CI: 1.07–1.30), respectively. Furthermore, these results were repeated in nested case–control analyses.

Ghaderi et al. (2024) (18) reported a 6% higher risk of PC for individuals in the highest versus lowest vitamin D category, although it was not statistically significant in the random-effects model (OR = 1.06; 95% CI: 0.98–1.14) and fixed-effects model (OR = 1.06; 95% CI: 0.99–1.14). Moreover, Gilbert et al. (2011) (a) (17), applying a random-effects model, observed a small positive association corresponding to an approximate 4% increase in PC risk; however, it was not statistically significant (OR = 1.04; 95% CI: 0.99–1.10).

#### 3.5.3 Association of vitamin D levels and prostate cancer incidence based on RR effect sizes

Three studies (four analytical arms) reporting RRs evaluated PC incidence using continuous dose–response exposure contrasts, primarily examining risk per 10 ng/mL increase in circulating 25(OH)D levels (4, 10, 19).

Yin et al. (2009) (19) reported no statistically significant association. In their fixed-effects analysis (Yin 2009 a) (19), each 10 ng/mL increase in 25(OH)D was associated with a 3% higher risk of PC (RR = 1.03; 95% CI: 0.98–1.09). The random-effects model (Yin 2009 b) (19) yielded a similar result (RR = 1.03; 95% CI: 0.96–1.10).

Similarly, Gandini et al. (2011) (10) found no evidence of an association, with a summary RR of 0.99 (95% CI: 0.95–1.03) per 10 ng/mL increase in 25(OH)D levels using a random-effects model.

In contrast, a low-quality study that was carried out by Gao et al. (2018) (4) reported a small statistically significant positive association. Their analysis found that each 10 ng/mL increase in 25(OH)D was associated with a 4% higher risk of PC (RR = 1.04; 95% CI: 1.02–1.06) based on a random-effects model. Notably, the highest versus lowest analysis showed a stronger positive association (RR = 1.15; 95% CI: 1.06–1.24).

### 3.6 Findings from the association of vitamin D with prostate cancer mortality

Meta-analyses focusing on mortality outcomes and on the 25(OH)D consistently reported null or inconsistent associations with PC outcomes. Notably, Cui et al. (2024) (21) reported an increased mortality risk associated with higher pretreatment 25(OH)D levels in a high-quality study (HR = 1.52; 95% CI: 1.26–1.83) (21). In contrast, a moderate quality by Song et al. (2018) (20) demonstrated a modest protective association, showing that each 20-ng/mL increase in circulating 25(OH)D concentration was associated with a 9% reduction in PC–specific mortality (HR = 0.91; 95% CI: 0.85–0.97) (20).

## 4 Discussion

The present umbrella review synthesizes findings from eight meta-analyses and 15 analytical arms evaluating whether circulating vitamin D biomarkers—primarily 25-hydroxyvitamin D [25(OH)D] and, to a lesser extent, 1,25-dihydroxyvitamin D [1,25(OH)_2_D]—are associated with PC risk or mortality. Across study designs and biomarker types, the evidence suggests a modest but positive association between higher circulating 25(OH)D levels and increased PC incidence, whereas data on mortality remain contradictory. However, the pooled OR of 1.06 is very modest and may be explained by residual confounding, detection bias, differential PSA screening practices, or healthcare utilization patterns rather than a true biological increase in risk. These findings collectively challenge traditional assumptions that vitamin D universally protects against cancer and indicate that its role in prostate carcinogenesis may be more biologically complex. Causality cannot be established from this observational umbrella review.

The strongest and most consistent evidence emerged from categorical high vs. low comparisons of 25(OH)D levels. Xu et al. (2014) (9) in a high-quality evaluation, reported a robust 17% increased risk of PC for men in the highest vitamin D category, a finding mirrored exactly in their nested case–control subgroup (9). The repetition of these results is consistent with the hypothesis that elevated vitamin D is linked to increased PC risk. In another high-quality assessment, Ghaderi et al. (2024) (18) further reported this association, reporting comparable effect estimates (both fixed and random models), though marginally non-significant (18). Collectively, categorical analyses rarely produced pooled effects below unity, indicating that higher vitamin D status may correspond with increased likelihood of PC diagnosis.

The overall pooled analysis in this umbrella review confirmed a statistically significant, though modest, elevation in PC risk. Sensitivity tests demonstrated near-identical pooled effects when individual studies were removed, indicating that these findings are not driven by a single influential meta-analysis. Heterogeneity remained low, consistency across studies despite geographic, demographic, and methodological variability.

Multiple biological potential mechanisms may explain why higher circulating 25-hydroxyvitamin D levels are likely associated with increased PC incidence, despite the protective roles that vitamin D exhibits in many other tissues (22, 23). Prostate cells may possess a distinct hormonal microenvironment, and the vitamin D metabolic pathway behaves differently within prostate tissue. One key mechanism involves dysregulation of the vitamin D receptor (VDR) signaling axis. Although vitamin D generally induces antiproliferative and pro-differentiation pathways, several studies have shown that prostate tumors may frequently overexpress CYP24A1, the enzyme responsible for degrading active 1,25-dihydroxyvitamin D (24, 25). CYP24A1 overexpression not only may diminish intracellular vitamin D activity but also likely promotes tumor progression by enabling cancer cells to evade vitamin D–mediated growth inhibition. Elevated circulating vitamin D may paradoxically increase substrate availability for CYP24A1-driven degradation, thereby plausibly amplifying tumor-promoting pathways within the prostate microenvironment (26, 27).

Another assumed mechanism involves androgen–vitamin D cross-talk. Androgen receptor (AR) signaling is a central driver of prostate carcinogenesis. Experimental studies demonstrate that vitamin D can upregulate AR expression or modify AR responsiveness under certain conditions, potentially enhancing androgen-mediated proliferation in early tumorigenesis (28). Conversely, in more advanced or androgen-deprived states, vitamin D may inhibit cancer progression, which might help explain why lower vitamin D predicts worse survival but higher vitamin D is associated with greater incidence. Additionally, genetic polymorphisms in VDR (such as FokI, BsmI, and TaqI) have been linked to altered PC susceptibility and may modify individual responses to vitamin D exposure. Men harboring “high-risk” VDR variants may experience increased proliferative signaling when exposed to higher vitamin D concentrations (29). All these mechanisms require confirmation in human clinical studies.

In contrast, continuous models examining incremental increases in circulating 25(OH)D failed to reproduce the associations observed in categorical analyses. For example, a high-quality study by Yin et al. (2009) (19) and a moderate one by Gandini et al. (2011) (10) found no meaningful association (10). But, only a low-quality study by Gao et al. (2018) (4) demonstrated a statistically significant slope, per 10 ng/mL increase in 25(OH)D (4). Remained confounders like sunlight exposure, physical activity, etc., may result in these outcome inconsistencies. Nonetheless, the combined continuous-effect model in this umbrella review yielded RR = 1.02 (95% CI: 0.98–1.06; p = 0.207), with an I² of nearly zero, supporting the conclusion that the relationship is not clearly linear.

An important observation in this umbrella review is the discrepancy between the categorical and continuous analyses. While the highest vs. lowest comparisons suggested a potential threshold or non-linear effects of vitamin D concentrations in PC increment risk, the dose–response analysis per 10 ng/mL increment did not show a statistically significant association; moreover, potential non-linear endocrine activity of vitamin D (30), may address this incoherency. One possible explanation is that categorical analyses may capture a threshold effect, whereas dose–response models assume a linear relationship between vitamin D and risk. This discrepancy suggests that the relationship between vitamin D and PC may not be linear and that risk might increase only after reaching higher physiological thresholds rather than through small incremental increases in serum concentration. Such threshold effects are biologically plausible in prostate tissue, where hormonal signaling pathways can exhibit nonlinear dose–response patterns. Additionally, differences in exposure categorization, study populations, and adjustment strategies across the included meta-analyses may also contribute to these contrasting findings; therefore, the observed discrepancy should be interpreted cautiously.

An important alternative explanation is that higher vitamin D levels may simply act as a surrogate marker for healthier individuals who undergo more frequent medical evaluation and PSA-based cancer detection, leading to surveillance bias. Additionally, reverse causation cannot be fully excluded, as subclinical disease could influence vitamin D metabolism or behavior. Confounding variables that may influence both vitamin D levels and PC detection include PSA screening frequency, obesity, metabolic syndrome, ethnicity, latitude, seasonal variation at blood draw, vitamin D supplementation behavior, physical activity, sunlight exposure, socioeconomic status, and healthcare access. Each of these factors could independently bias the observed associations, and residual confounding cannot be eliminated in observational umbrella reviews.

A major theme emerging from this review is the importance of study design. NCC studies consistently demonstrated significant associations between elevated vitamin D levels and increased PC risk, although these studies have limitations, such as changes in potential confounders like lifestyle habits over time, they are less vulnerable to detection bias and reverse causation because vitamin D levels are measured before clinical diagnosis. Prospective cohort studies exhibited similar patterns, though with slightly more variation in significance. In contrast, analyses that merged case–control and cohort studies into a single pooled estimate often yielded non-significant results, highlighting the limitations of combining designs with fundamentally different exposure timing and measurement accuracy. When the highest-quality designs were prioritized, the associations became clearer, stronger, and more consistent.

This umbrella review has multiple strengths. It integrates results from the highest-quality meta-analyses available, applies sensitivity testing, incorporates AMSTAR-2 quality assessment, and synthesizes evidence across incidence and mortality outcomes. The analytical arms include tens of thousands of participants, improving precision and generalizability. The review also highlights consistent patterns across biomarker type, model structure, and study design—patterns that individual meta-analyses often could not evaluate alone.

Nevertheless, several limitations must be acknowledged. Vitamin D assays differ substantially across studies, introducing measurement heterogeneity. Confounding from sunlight exposure, physical activity, BMI, diet, and socioeconomic status remains difficult to eliminate. Categorical definitions of “high” and “low” vitamin D vary widely, complicating comparisons. Geographic differences—such as the contrasting results from Finland and South Korea observed in Ghaderi (2024) (18)—suggest that genetic, environmental, geographic coordinates variations, which cause different radiation intensity of the sun, or lifestyle factors may modify associations. Mortality analyses often rely on a single baseline vitamin D measurement, which may not reflect long-term exposure or disease progression.

Given these findings, clinical implications must be interpreted cautiously. While vitamin D supplementation remains essential in most biological pathways, no evidence supports its use for PC prevention, and emerging data from this umbrella review raise concern that high circulating levels could modestly increase PC risk. This is particularly relevant in older men and those using high-dose vitamin D supplements. Conversely, inadequate vitamin D may worsen outcomes after PC diagnosis, suggesting a hypothesis of a potential U-shaped risk curve. Personalized vitamin D assessment by following clinical guidelines, rather than indiscriminate supplementation, may therefore be more appropriate for men at risk of or living with PC.

Future research should include, first of all, standardized, repeated vitamin D measurements to compare its level based on the same metric, careful adjustment for relevant confounders, and stratification by tumor aggressiveness. Mendelian randomization could help clarify causality, and randomized trials of supplementation by considering the possibility of harms suggested in observational data and medical ethics to prevent PC initiation.

## 5 Conclusion

This umbrella review provides evidence that higher circulating 25(OH)D levels are slightly associated with increased PC incidence, in categorical analyses and nested case–control studies. No linear dose–response pattern was detected in continuous models, suggesting a potential threshold effect. The active metabolite 1,25(OH)_2_D showed no association with risk that may originate from the limited number of included studies (only one). Mortality findings remain inconsistent. Together, these results indicate that vitamin D biology in PC may be complex and potentially is distinct from its role in other malignancies, underscoring the need for cautious interpretation. Due to limitations like different vitamin D assay methodologies, various thresholds, and probable detection bias, we recommend utilizing standardized assays, repeated measurements, and Mendelian randomization design in future investigations.

## Data Availability

The data analyzed in this study are derived from previously published studies. Therefore, no new datasets were generated. All relevant data are included in the article and its **Supplementary Material**, and the original sources are cited in the reference list.
