# Supplementary material for: Serum vitamin D levels and prostate cancer: an umbrella review and pooled analysis of observational meta-analyses
Source: Front Oncol. 2026 Jun 29;16:1798697. doi: 10.3389/fonc.2026.1798697 (PMC13357282; doi:10.3389/fonc.2026.1798697)

**Supplementary Figure 1.** Leave-one-out sensitivity analysis for exploring the association of highest versus lowest levels of circulating vitamin D levels with odds of prostate cancer occurrence


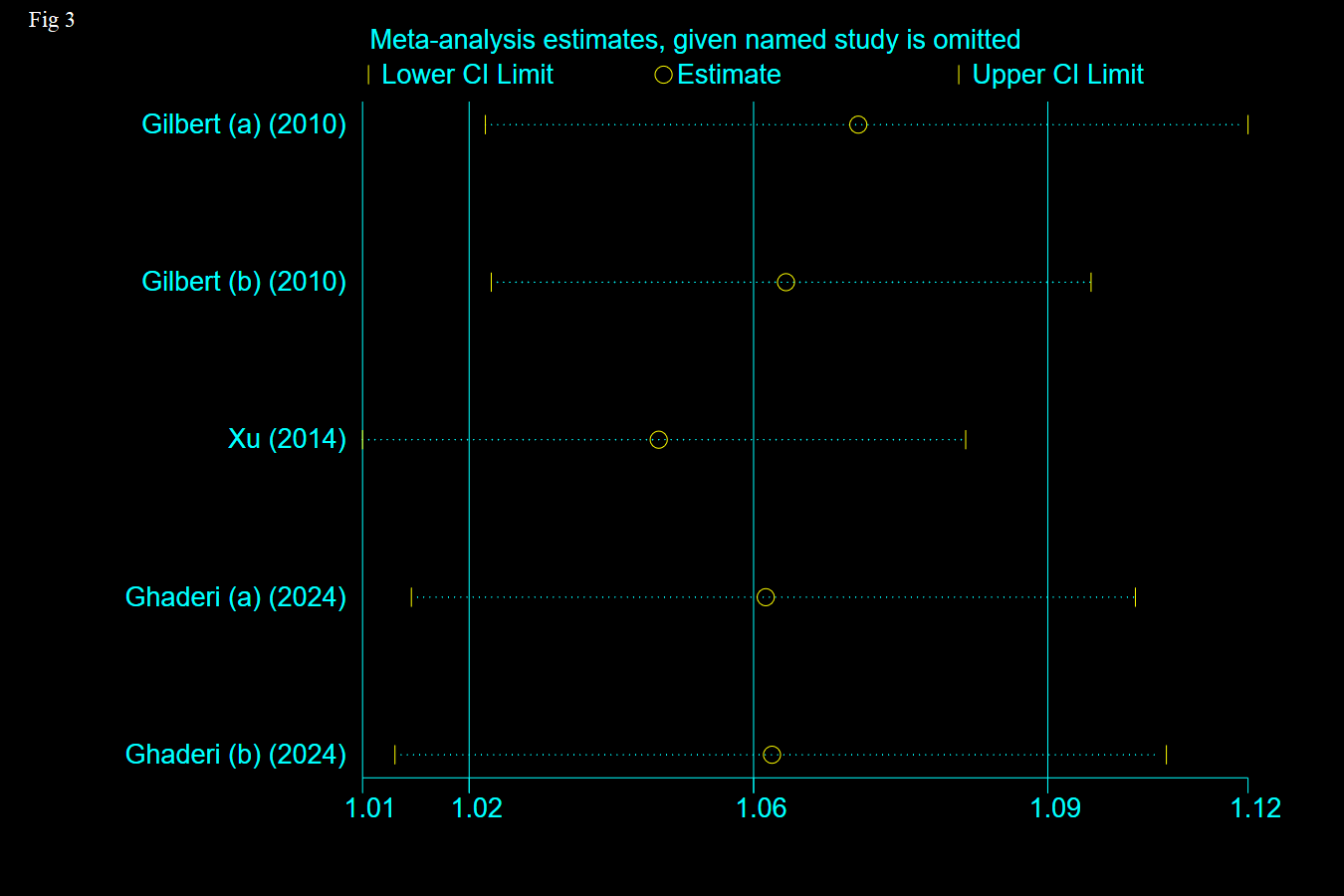


**Supplementary Figure 2.** Leave-one-out sensitivity analysis for exploring the association of vitamin D levels on odds of prostate cancer occurrence per 10-ng/mL increase in circulating 25(OH)D.


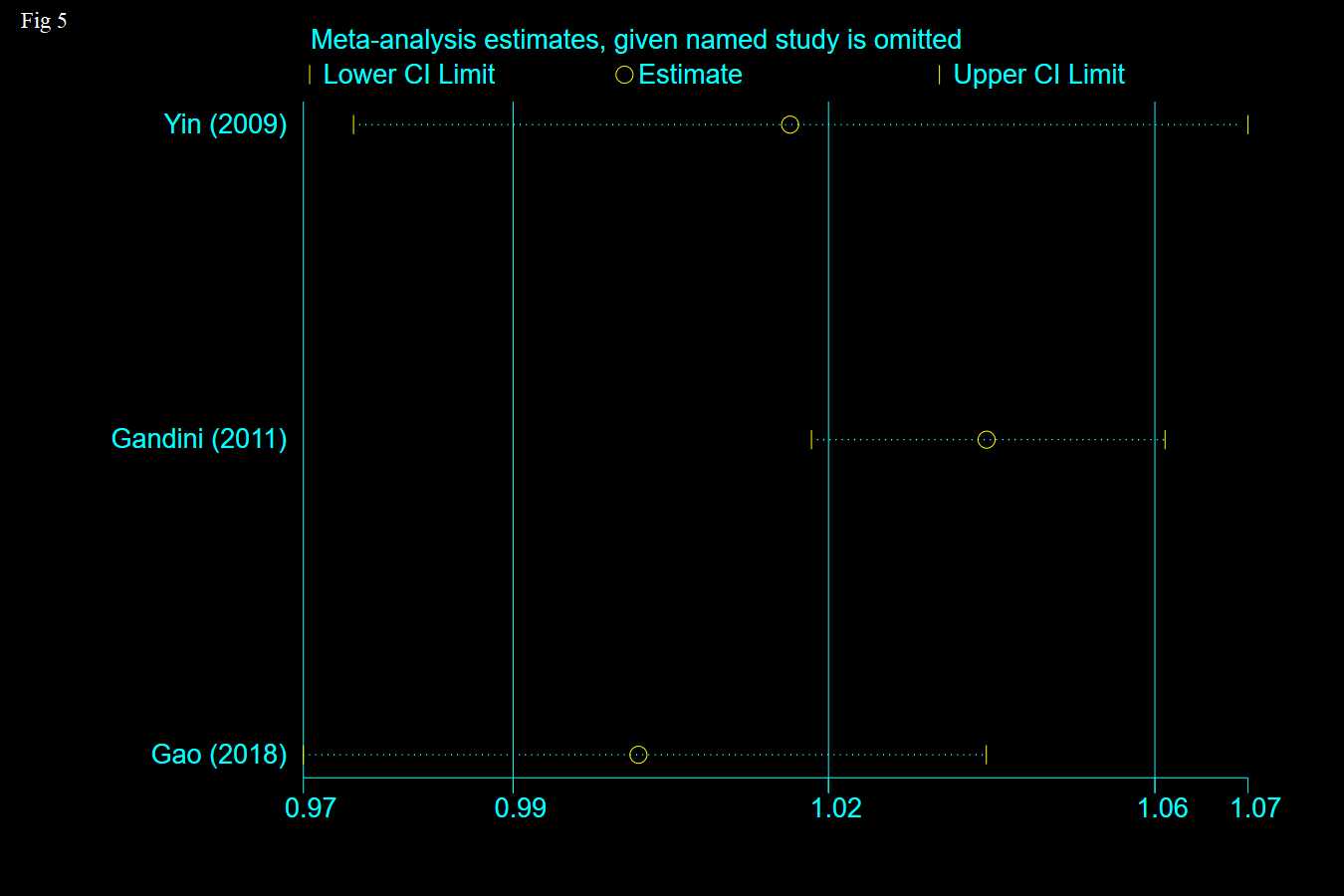

Supplement: Supplementary file 1 [file DataSheet1.docx]
