# Supplementary material for: Serum vitamin D levels and prostate cancer: an umbrella review and pooled analysis of observational meta-analyses
Source: Front Oncol. 2026 Jun 29;16:1798697. doi: 10.3389/fonc.2026.1798697 (PMC13357282; doi:10.3389/fonc.2026.1798697)
Supplement: Supplementary file 2 [file DataSheet2.docx]

| Terms | | |
| --- | --- | --- |
| "Vitamin D" OR "Ergocalciferols" OR "Vitamin D Deficiency" OR "Cholecalciferol" OR Vitamin D OR Ergocalciferols OR Cholecalciferol OR "25-hydroxycholecalciferol" | "prostatic neoplasms" OR "prostate cancer" OR "prostate tumor" OR "prostatic tumor" OR "Prostatic Neoplasms" | meta-analysis OR meta analysis |

Supplementary Table 1.

|  | Search strategy | number |
| --- | --- | --- |
| PubMed | ((((("Vitamin D"[Mesh] OR "Ergocalciferols"[Mesh] OR "Vitamin D Deficiency"[Mesh] OR "Cholecalciferol"[Mesh]) OR (((Vitamin D[Title/Abstract]) OR (Ergocalciferols[Title/Abstract])) OR (Cholecalciferol[Title/Abstract])) OR ("25-hydroxycholecalciferol"[Title/Abstract])))) AND ((((("prostatic neoplasms"[Title/Abstract]) OR ("prostate cancer"[Title/Abstract])) OR ("prostate tumor"[Title/Abstract])) OR ("prostatic tumor"[Title/Abstract])) OR ("Prostatic Neoplasms"[Mesh]))) AND ((meta-analysis[Publication Type]) OR (meta analysis[Title/Abstract])) | 47 |
| Isi web of science | #1 AND #2 AND #3 | 143 |
| Scopus | ( ( TITLE-ABS-KEY ( vitamin D ) OR TITLE-ABS-KEY ( "Vitamin D Deficiency" ) OR TITLE-ABS-KEY ( cholecalciferol ) OR TITLE-ABS-KEY ( "25-hydroxycholecalciferol" ) OR TITLE-ABS-KEY ( Ergocalciferol ) ) ) AND ( ( TITLE-ABS-KEY ( "prostatic neoplasm" ) OR TITLE-ABS-KEY ( "Prostate neoplasm" ) OR TITLE-ABS-KEY ( "prostate cancer" ) OR TITLE-ABS-KEY ( "prostate tumor" ) OR TITLE-ABS-KEY ( "prostatic tumors" ) ) ) AND ( ( TITLE-ABS-KEY ( meta analysis ) OR TITLE-ABS-KEY ( meta-analysis ) ) ) | 187 |
| All |  | 377 |
| Duplicate |  | 98 |
